# Research quality and transparency, outcome measurement and evidence for safety and effectiveness in robot‐assisted surgery: systematic review

**DOI:** 10.1002/bjs5.50352

**Published:** 2020-10-14

**Authors:** P. Garfjeld Roberts, J. C. Glasbey, S. Abram, D. Osei‐Bordom, S. P. Bach, D. J. Beard

**Affiliations:** ^1^ Nuffield Department of Orthopaedics, Rheumatology and Musculoskeletal Sciences University of Oxford UK; ^2^ Royal College of Surgeons Surgical Intervention Trials Unit Oxford UK; ^3^ Academic Department of Surgery UK; ^4^ Diagnostics, Drugs, Devices and Biomarkers (D3B) and University of Birmingham Birmingham UK; ^5^ Royal College of Surgeons of England London UK

## Abstract

**Background:**

Robot‐assisted surgery (RAS) has potential panspecialty surgical benefits. High‐quality evidence for widespread implementation is lacking. This systematic review aimed to assess the RAS evidence base for the quality of randomized evidence on safety and effectiveness, specialty ‘clustering’, and outcomes for RAS research.

**Methods:**

A systematic review was undertaken according to PRISMA guidelines. All pathologies and procedures utilizing RAS were included. Studies were limited to RCTs, the English language and publication within the last decade. The main outcomes selected for the review design were safety and efficacy, and study purpose. Secondary outcomes were study characteristics, funding and governance.

**Results:**

Searches identified 7142 titles, from which 183 RCTs were identified for data extraction. The commonest specialty was urology (35·0 per cent). There were just 76 unique study populations, indicating significant overlap of publications; 103 principal studies were assessed further. Only 64·1 per cent of studies reported a primary outcome measure, with 29·1 per cent matching their registration/protocol. Safety was assessed in 68·9 per cent of trials; operative complications were the commonest measure. Forty‐eight per cent of trials reported no significant difference in safety between RAS and comparator, and 11 per cent reported RAS to be superior. Efficacy or effectiveness was assessed in 80·6 per cent of trials; 43 per cent of trials showed no difference between RAS and comparator, and 24 per cent reported that RAS was superior. Funding was declared in 47·6 per cent of trials.

**Conclusion:**

The evidence base for RAS is of limited quality and variable transparency in reporting. No patterns of harm to patients were identified. RAS has potential to be beneficial, but requires continued high‐quality evaluation.

## Graphical abstract

Robot‐assisted surgery is expanding rapidly, but the evidence base is poor. This article demonstrates the heavy weighting to research in certain specialties, no clear safety or efficacy benefit, inconsistent choice of outcome measures, and poor research governance. These factors significantly increase the risk associated with adopting these potentially revolutionary technologies.

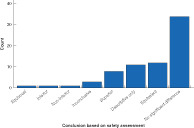

Call for better quality evidence.

## Abstract (es)

**Antecedentes:**

La cirugía robótica (*robotic‐assisted surgery*, RAS) ofrece beneficios en muchas especialidades quirúrgicas. Se carece de evidencia de alta calidad para una implementación generalizada de la cirugía robótica. Esta revisión sistemática tenía como objetivo evaluar las bases de la evidencia para llevar a cabo investigaciones sobre seguridad y efectividad basadas en diceños aleatorizados, agrupación de especialidades quirúrgicas y resultados de la RAS.

**Métodos:**

Se realizó una revisión sistemática siguiendo las recomendaciones PRISMA. Se incluyeron todas las patologías y procedimientos que utilizaban RAS. Los estudios se limitaron a ensayos clínicos controlados (*randomised controlled trials*, RCTs), publicados en inglés y en la última década. Los principales resultados seleccionados para el diseño de la revisión fueron la seguridad y la eficacia, y el propósito del estudio. Los resultados secundarios fueron las características del estudio, la financiación y la gobernanza.

**Resultados:**

Se identificaron 7.142 artículos de los que se seleccionaron 183 RCTs para la extracción de datos. La especialidad más frecuente era urología (35,0%). Solo hubo 76 poblaciones de estudio únicas, lo que suponía una superposición significativa de publicaciones, por lo que se evaluaron 103 estudios principales. Solamente un 64,1% de los estudios publicaron datos de la variable principal, con un 29,1% coincidiendo con el registro/protocolo correspondiente. La seguridad se evaluó en el 68,9% de los ensayos, siendo las complicaciones operatorias la variable analizada con más frecuencia. La mayoría de los ensayos (47,9%) demostraban que no había diferencias significativas en la seguridad entre RAS y el procedimiento comparador, aunque un 11,3% señalaban que RAS era superior. En un 80,6% se había evaluado la eficacia o la efectividad; un 43,4% de los ensayos no mostraban diferencia entre RAS y el comparador, mientras que en un 24,1% RAS era superior. En un 47,6% de los ensayos se declaraba las fuentes de financiación.

**Conclusión:**

La base de la evidencia de RAS es de calidad limitada y la transparencia en la comunicación de los resultados es variable. Respecto a la seguridad, no se han identificado patrones dañinos para los pacientes. RAS tiene el potencial de ser beneficioso, pero precisa ser objeto de evaluación continuada de alta calidad.

## Introduction

Robot‐assisted surgery (RAS) has undergone rapid development in the past 15 years. It has been approved by regulators worldwide across most surgical specialties for a wide range of surgical indications. For some procedures RAS has become standard of care, for example in prostatectomy^1^. There has also been substantial uptake of RAS, particularly in North America, for a number of procedures where the evidence base seems absent or inconsistent, but driven by patient demand, industry and functional benefits for surgeons^2^.

Currently RAS consists of a ‘master–slave’ model in which an ‘active’ system reproduces the surgeon's actions by performing discrete tasks under the control of the surgeon^3^. The proposed robotic value‐proposition includes the potential for remote surgery, and mirrors many benefits observed for minimal‐access surgery, such as less scarring, reduced physiological insult of surgery, shorter length of stay and faster recovery time. It has also been proposed that surgical precision may be improved, for example in implant positioning^4^ or surgical resection margins^5^. There may be greater safety, fewer complications^6^ and better access than that achieved by traditional minimally invasive surgery (MIS)^7^. There may be haptic benefits, with robotically assisted microsurgery able to detect and control previously undetectable forces to minimize intraocular injury in eye surgery^8^, and robotic surgery may have a shorter learning curve than an equivalent standard minimal‐access technique^4^.

Despite early and anecdotal evidence of potential benefit, the innovation is yet to be fully explored and documented. It is important that safety is maintained during early implementation, and that effectiveness compared with open and traditional MIS techniques is demonstrated to justify investment in platforms and training. Robotic surgical platforms are costly in terms of capital expenditure, infrastructure adaptation, and ongoing use of consumables^9^, ^10^.

Randomized trials are the current foundation of this evidence base. RCTs are an important component of evaluation of innovation as they have the lowest risk of bias. This maps to the IDEAL (Idea, Development, Exploration, Assessment, Long‐term study) model of surgical evaluation in which evidence is collected systematically and documented in an appropriate sequence^11^. RCTs represent stage 3 of this pathway, where the innovation platform is stable but lacks evidence of comparative effectiveness to support wider implementation.

This review aimed to understand the general state of current safety and efficacy evidence across the RAS field. This will directly guide research questions and prioritization^12^. The primary aim of the study was to document the clinical aims and selected metrics from RCTs regarding the safety and efficacy of RAS across surgical specialties. Secondary aims were to explore the breadth of outcome reporting in the context of increasing uptake of RAS. A final aim was to explore the quality of research design and governance for RAS‐related research.

## Methods

A review of the literature was conducted according to PRISMA guidelines^13^. Study aims, design and search strategy were specified using the participants, interventions, comparators, outcomes and study design (PICO) process.

### Protocol and registration

This systematic review was registered with PROSPERO (registration number CRD42019046621). There have been no changes to the protocol registered with PROSPERO. The protocol has not been published separately.

**Fig. 1:**
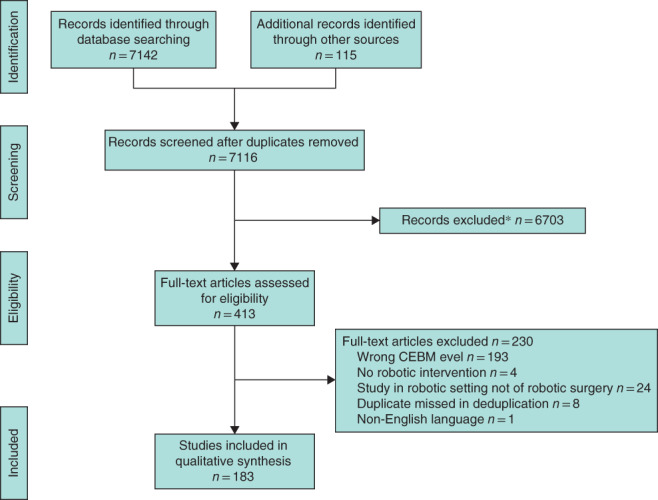
PRISMA flow diagram for the review *Some 1426 of these records were retained separately for inclusion in a pansurgical robot‐assisted surgery database. CEBM, Centre for Evidence‐Based Medicine.

### Eligibility criteria

A search strategy was devised with the assistance of an expert librarian using a PICO framework. Studies of patients of any age with pathology that could be addressed by a RAS procedure were eligible. Studies comparing RAS with open, endoscopic or an alternative RAS procedure were included, as were comparators between RAS with an alternative medical or conservative treatment, or no treatment at all. Outcomes were related to procedural safety measures (such as complication rate, conversion to open procedure) and efficacy/effectiveness (for example, resection margin status, implant positioning, functional or patient‐reported outcome measures (PROMs), length of stay, duration of surgery). Only trials published in the English language were included.

### Information sources

A comprehensive search of the Ovid MEDLINE, Ovid Embase, Cochrane CENTRAL, Scopus and Web of Science Core Collection was conducted from 1 January 2008 to 23 August 2019. The search strategy was designed to maximize the numbers of references retrieved about RCTs involving RAS. These were then screened for eligibility against the protocol inclusion criteria. The search strategies used free‐text words and combinations of the relevant thesaurus terms. Further relevant studies were identified from the reference and citation lists of all included articles, from reference lists of systematic reviews, from published protocols for planned or ongoing trials, and from online registries for RCTs. The full search strategies for each database, using equivalent search terms for the database subheading mappings, are included in *Appendix* *S1* (supporting information).

### Study selection

Studies identified by the search strategy were imported into Covidence, the Cochrane Collaboration's online systematic review management platform, which performed automatic deduplication. Each of the remaining titles and abstracts was screened independently and in duplicate by two of five reviewers familiar with the study aims and protocol. Screened studies underwent full‐text review in duplicate, and were selected for inclusion in this systematic review according to the inclusion and exclusion criteria (*Table* *1*).

**Table 1:** Detailed study inclusion and exclusion criteria

Inclusion criteria	Exclusion criteria
Robotically assisted/performed procedures on live humans Cadaveric studies where a measure of safety, efficacy or accuracy is primary outcome RCTs of robotically assisted surgery *versus* at least one other form of treatment for the condition	Inactive/non‐robotic computer assistance, such as navigation, templating, positioning Comparisons between solely alternative robotic techniques Opinion pieces or editorials Proof‐of‐principle studies Animal or dry bone studies Studies in robotic setting, not of robotic surgery (e.g. anaesthetic techniques) Non‐English language Interventional radiology or medical robotics

### Data collection process

From the articles selected for inclusion, data were extracted using an online, standardized, collaborative data extraction platform using Google Sheets™ (Google, Menlo Park, California, USA). Data extraction was piloted on a subset of the search results to optimize the data extraction process.

### Study assessment characteristics (PRISMA outcomes)

The main areas of interest for this review (primary outcome measures in terms of PRISMA design) were the stated purpose of each study and reported safety and efficacy/effectiveness outcomes, including description of the outcome measures used. Secondary areas of interest (secondary PRISMA outcome measures) included study quality characteristics, design, funding and research governance features. PRISMA outcome definitions and how each was coded can be found in *Table* *2*. Safety was taken to mean the treatment‐attributable harms, but not risks of harm (such as surgical time, tourniquet time without recorded harmful event) or differences in long‐term disease‐related outcomes (such as recurrence rates for oncological procedures, or revisional surgery rates). Efficacy and effectiveness were defined as measures related to the indication for the procedure and their main clinical outcomes. For included trials, the relevant clinical trials registry submission or published protocol was cross‐referenced to identify differences between the planned and reported primary analyses.

**Table 2:** Extraction fields and descriptions for outcomes of selected studies

Outcome recorded from study	Description
**Main purpose of study**	
Primary outcome	What the stated primary outcome of interest was in the study report; otherwise recorded as ‘none listed’
Matches trial registration	Whether primary outcome matches that in trial registration
*A priori* power	Whether the study was powered to the primary outcome, another outcome, or no power calculation provided
‘Main’ outcome	If no primary outcome stated, a subjective assessment of the finding given most weight in the report
Purpose of primary or main outcome	Coded as: Clinician assessment (findings on clinical examination); PROMs; functional outcome (objective measure of organ or patient activity); process outcome (measures of perioperative systems that work to deliver a surgical outcome); complications (general or procedure‐specific perioperative adverse events); oncological (measures of oncological activity, recurrence and survival); precision (measurement of location or accuracy relative to a recognized standard)
**Safety and efficacy assessments**	
Presence	True/false; whether an outcome related to safety or efficacy was recorded
Measure	Measures used to report safety/efficacy
Follow‐up period	Length of follow‐up; time in months, or descriptor if not stated
Analysis design	The statistical approach to assessing outcomes: superiority, equivalence, non‐inferiority, unclear, or descriptive only
Conclusion	The conclusion based on the safety or efficacy measures listed: superior, inferior, non‐inferior, equivalent, no significant difference, or descriptive only (NB: regarding safety/efficacy outcome only, not the overall conclusions of the report)
**Study characteristics and design**	
Specialty	Surgical specialty
Study ID	Trial registry number, or generated identifier if not registered, to consolidate multiple reports from single study population
Nature of report	Preliminary report (any report before planned trial endpoint); main report (primary outcomes at planned trial endpoint); secondary outcomes (secondary outcomes at planned trial endpoint); long‐term follow‐up (any report after planned trial endpoint)
Pathology addressed	Surgical pathology that is indication for surgery
Robotic device	Name of device and manufacturer
No. of groups	No. of comparison groups in the trial
Participants	No. of participants in robotic, MIS, open and non‐operative arms
Procedure	Name of robotic procedure, and whether MIS/open/non‐operative intervention was an equivalent procedure
**Secondary outcomes**	
List of outcomes	Complete list of the outcomes assessed in a study
No. of outcomes	Total no. of outcomes assessed in a study; if an outcome is assessed at multiple time points, each time point counts towards this. If a report subclassifies outcomes for statistical assessment (e.g. subscales within a PROM), each of these is recorded as an outcome
**Research governance**	
Prospective registration	True/false; whether the study was registered prospectively
Funding declaration	True/false; whether the study included a funding declaration
COI declaration	True/false; whether the study included a COI declaration

PROM, patient‐reported outcome measure; MIS, minimally invasive surgery; COI, conflict of interest.

### Risk of bias in individual studies

Risk of bias was not assessed in studies using a formal tool as no meta‐analysis was planned. However, important research governance and study design features were recorded during data extraction (*Table* *2*). A high degree of heterogeneity was anticipated between studies as it was planned to include studies from multiple procedure types across all surgical specialties, with descriptive reporting of the findings to help understand the evidence available and guide further research. As such, no synthesis of data or results was planned or expected to be possible, with no formal assessment of bias within studies.

In response to the issue of multiple publications from the same study populations, a further analysis was conducted to account for the repeat reporting. Studies were divided according to the ‘nature of the report’ (*Table* *2*). ‘Preliminary studies’ before the trial end date were reviewed and analysed further only if they were the most recently published report. All ‘main reports’ were included within further analysis (duplicate publications in separate journals were excluded). Only the most recent time point of ‘long‐term follow‐up’ of primary outcomes for a given study population received further attention and inclusion.

## Results

The search of Ovid MEDLINE, Ovid Embase, Cochrane CENTRAL, Scopus and the Web of Science Core Collection identified a total of 7142 titles. After deduplication, 7116 unique titles and abstracts were screened by two reviewers independently: 6703 were excluded by both reviewers or after conflicts had been resolved by the lead author. Of 413 titles that were put forward for full‐text review, 183 RCTs were identified for data extraction. Study selection is summarised in *Fig*. *1*.

### Study characteristics

Of the 183 trials identified, the commonest specialty group was urology (64 studies; 35·0 per cent), followed by obstetrics and gynaecology (50 studies; 27·3 per cent) and general surgery (22 colorectal and 13 upper gastrointestinal studies; 19·1 per cent). No RCTs were identified for breast, plastic, neurosurgery, oral and maxillofacial, otolaryngology or vascular surgery (*Table* *3*). The commonest pathologies addressed were bladder cancer (29 of 183, 15·8 per cent), pelvic organ prolapse (22 of 183, 12·0 per cent) and prostate cancer (19 of 183, 10·4 per cent) (*Table* *4*).

**Table 3:** Representation of surgical specialties in identified studies

Specialty	No. of studies (*n* = 183)
Urology	64
Obstetrics and gynaecology	50
Trauma and orthopaedics	27
Colorectal	22
Upper gastrointestinal	13
Cardiac	2
Endocrine	1
Ear, nose and throat	1
Paediatrics	1
Thoracic	1
Transplant	1

**Table 4:** Pathology addressed or specific procedure performed in methods of included studies

Pathology addressed or procedure	No. of studies (*n* = 183)
Bladder malignancy	29
Pelvic organ prolapse	22
Prostate malignancy	19
Benign prostatic hypertrophy	12
Rectal malignancy	12
Hysterectomy (for benign or malignant indications)	11
Endometrial malignancy	8
Right‐sided colorectal malignancy	6
Benign gallbladder disease	5
Lumbar spinal stenosis	5
Osteoarthritis of hip	5
Osteoarthritis of knee	5
Cervical malignancy	4
Osteoarthritis of medial compartment of knee	4
Benign surgical spinal pathology	3
Oesophageal malignancy	3
Endometriosis	3
Gastric malignancy	2
Colorectal malignancy with liver metastases	2
Degenerative spinal disorder	2
Incisional hernia	2
Pancreatectomy (benign/low‐grade malignant indication)	2
Renal malignancy	2
Benign thyroid nodule	1
Uterine, cervical or ovarian malignancy	1
Cardiac bypass	1
Cardiac pathology requiring median sternotomy	1
Gastro‐oesophageal reflux disease	1
Kidney donation	1
Lumbosacral spinal disorders	1
Lung malignancy	1
Obesity with renal failure	1
Oropharyngeal squamous cell carcinoma	1
Pelvic ring posterior instability	1
Thoracolumbar spinal disorder	1
Hysterectomy ± salpingo‐oophorectomy (for benign or malignant indication)	1
Ureteropelvic junction obstruction	1
Urinary tract stones	1

**Fig. 2:**
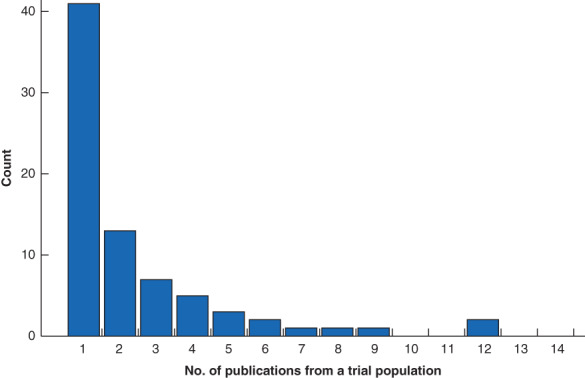
Frequency of publications identified from the 76 discrete randomized trials

Review of the study identifiers indicated that, of the 183 trials, patients were drawn from only 76 unique study populations. Two separate RAS trials in urological surgery generated up to 12 publications each from single populations (*Fig*. *2*).

Using only the most recent time point of ‘long‐term follow‐up’ of primary outcomes for a given study population yielded 103 principal studies^14^, ^15^, ^16^, ^17^, ^18^, ^19^, ^20^, ^21^, ^22^, ^23^, ^24^, ^25^, ^26^, ^27^, ^28^, ^29^, ^30^, ^31^, ^32^, ^33^, ^34^, ^35^, ^36^, ^37^, ^38^, ^39^, ^40^, ^41^, ^42^, ^43^, ^44^, ^45^, ^46^, ^47^, ^48^, ^49^, ^50^, ^51^, ^52^, ^53^, ^54^, ^55^, ^56^, ^57^, ^58^, ^59^, ^60^, ^61^, ^62^, ^63^, ^64^, ^65^, ^66^, ^67^, ^68^, ^69^, ^70^, ^71^, ^72^, ^73^, ^74^, ^75^, ^76^, ^77^, ^78^, ^79^, ^80^, ^81^, ^82^, ^83^, ^84^, ^85^, ^86^, ^87^, ^88^, ^89^, ^90^, ^91^, ^92^, ^93^, ^94^, ^95^, ^96^, ^97^, ^98^, ^99^, ^100^, ^101^, ^102^, ^103^, ^104^, ^105^, ^106^, ^107^, ^108^, ^109^, ^110^, ^111^, ^112^, ^113^, ^114^, ^115^, ^116^ for detailed analysis (*Table* *S1*, supporting information). There were 99 two‐arm studies (96·1 per cent) and four three‐arm studies (3·9 per cent), with a median of 78 (range 6–1516) patients. Control groups were MIS in 56 studies (54·4 per cent), open surgery in 48 studies (46·6 per cent) and non‐operative in three studies (2·9 per cent). The control group received an equivalent surgical procedure to the robotic group in 92 of the 103 studies (89·3per cent).

From these principal trials, the da Vinci® system (Intuitive Surgical, Sunnyvale, California, USA) was the commonest robotic system where this was specified in the reports (34 of 76, 45 per cent) (*Table* *5*), followed by Robodoc® (THINK Surgical, Freemont, California, USA) (6 of 76, 8 per cent). The device was unidentifiable in 25 study populations (33 per cent) from either the paper or the registration.

**Table 5:** Robotic device used in the 76 unique study populations

Robotic device	No. of studies*
Da Vinci® (Intuitive Surgical, Sunnyvale, California, USA)	34
Robodoc® (THINK Surgical, Freemont, California, USA)	6
Renaissance™ (Mazor Robotics, Caesarea, Israel)	2
TiRobot® (TiNavi Medical Technologies, Beijing, China)	2
SpineAssist® (Mazor Robotics)	2
AquaBeam® (Procept BioRobotics, Redwood Shores, California, USA)	1
AESOP® (Computer Motion, Santa Barbara, California, USA)	1
Avicenna Roboflex™ (Elmed Medical Systems, Ankara, Turkey)	1
ORTHODOC® (Integrated Surgical Technology, Wilmington, Delaware, USA)	1
RIO™ Robotic Arm Interactive Orthopedic System (MAKO Surgical, Davie, Florida, USA)	1

* The robotic device used was not reported in 25 principal studies.

### Primary and main outcomes

A primary outcome was declared formally in nearly two‐thirds of studies (66 of 103, 64·1 per cent), but only 29·1 per cent (30 of 103) matched a reported primary outcome listed in the protocol or a clinical trial registry. Some 43 different primary or main outcome measures were reported; these were highly heterogeneous and often specific to procedures or pathologies. The commonest primary or main reported outcome was duration of surgery (17 of 103, 16·5 per cent), with the majority (28 of 43, 65 per cent) being unique to a given study (*Tables* *6* and *7*). The interpreted purpose of the primary or main outcome was most frequently a process outcome (28 of 103, 27·2 per cent) or a PROM (25 of 103, 24·3 per cent) (*Table* *8*). About one‐third of studies (34 of 103, 33·0 per cent) were powered to detect a difference in the primary outcome measure, one‐half had no formal power calculation (55 of 103, 53·4 per cent) and the remainder were powered for another measure.

**Table 6:** Primary outcome variable formally reported in study methods

Primary outcome variable	No. of studies (*n* = 103)
None listed or powered for	37
Complication rate	7
Accuracy of implant position	7
Duration of surgery	6
Cost of surgery	4
Length of stay	4
Postoperative pain	4
Anatomical outcome	3
Disease‐free survival	3
International Prostate Symptom Score change	3
Continence	2
Cosmesis/self‐esteem	2
Japanese Orthopaedic Association Scale score	2
Lymph node retrieval	2
Secondary outcomes study	2
Expanded Prostate Cancer Index Composite (EPIC) urinary function score	2
Oswestry Disability Index	1
Conversion to open/abandon procedure	1
Erectile function	1
Functional recovery (suitability for discharge)	1
Implant survivorship	1
Insulin resistance	1
Knee Society Score	1
Quality of life, general scoring system	1
Quality of life, swallowing related (MD Anderson Dysphagia Inventory)	1
Quality of total mesorectal excision (pathologist graded)	1
Recurrence rate	1
Renal function	1
Surgeon stress/workload	1

**Table 7:** Main outcome variable of studies where no primary variable reported

Main outcome where no primary outcome stated	No. of studies (*n* = 37)
Duration of surgery	11
Cosmesis/self‐esteem	3
Accuracy of implant position	4
Lymph node retrieval	2
American Knee Society Score	1
Anatomical outcome assessment	1
Biochemical recurrence	1
Blood loss	1
Cost analysis	1
Finite element analysis of disc pressures	1
Gait kinematics	1
Graft patency	1
Overall survival	1
Oxford Knee Score	1
Positive surgical margin	1
Quality of life, general scoring system	1
Quality of Life in Reflux and Dyspepsia (QOLRAD)	1
Range of motion	1
Symptom score	1
UCLA grade	1
Weight loss	1

UCLA, University of California, Los Angeles.

**Table 8:** Interpretation of the purpose of primary or main reported outcome in included studies

Purpose of outcome	No. of studies (*n* = 103)
Process outcome	28
Clinical score/PROM	25
Oncological	11
Precision	9
Complication, general	8
Functional outcome	6
Clinician assessment	4
Radiological	4
Surgical access	2
Biochemical	2
Biomechanical	2
Complication, procedure‐specific	1
Surgeon factors	1

PROM, patient‐reported outcome measure.

### Safety assessment

**Fig. 3:**
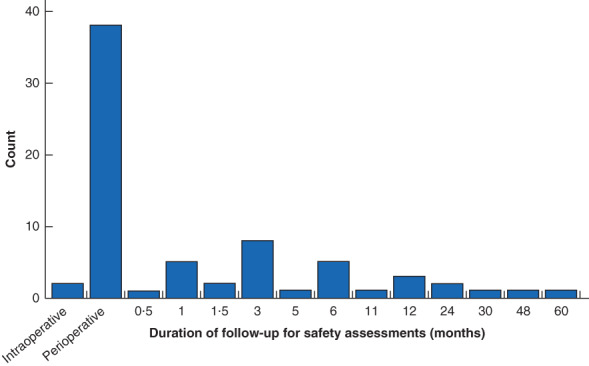
Frequency of follow‐up duration for the 71 studies that included safety assessment

**Fig. 4:**
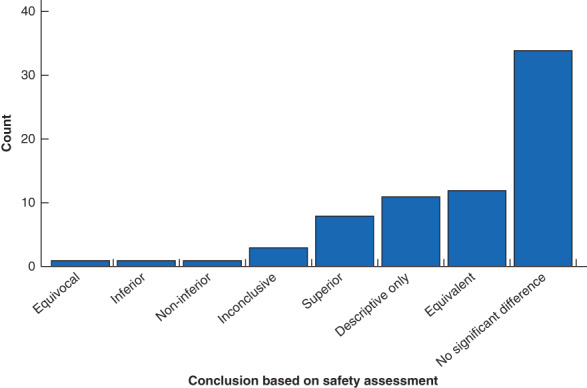
Conclusions based on safety parameters for the 71 studies that included safety assessment

Safety of robotic surgery was assessed in 68·9 per cent of the studies (71 of 103), although not necessarily as the primary or main outcome of interest. Where safety was assessed, the commonest measures were complications (70 of 71, 99 per cent), conversion to open surgery/alteration of surgical plan (32 of 71, 45 per cent), and the need for secondary or further intervention (28 of 71, 39 per cent). Blood loss was reported in 69 per cent of studies (49 of 71), but only 17 per cent (12 of 71) reported transfusion rate, and none reported the rate of transfusion‐related complications. The majority of these studies focused on the short term, with ‘perioperative’ being the commonest follow‐up period reported in studies, without further qualification (38 of 71, 54 per cent). Safety follow‐up of 1 year or more was included in only 11 per cent of studies (8 of 71) (*Fig*. *3*). Study conclusions based on the safety assessment most commonly reported no significant difference between the robotic surgery and control group (34 of 71, 48 per cent) or equivalence between the groups (12 of 71, 17 per cent). No studies were designed as equivalence trials or powered to make this conclusion. Robotic surgical outcomes were reported to be superior with regard to safety measures in 11 per cent of studies (8 of 71) (*Fig*. *4*).

**Fig. 5:**
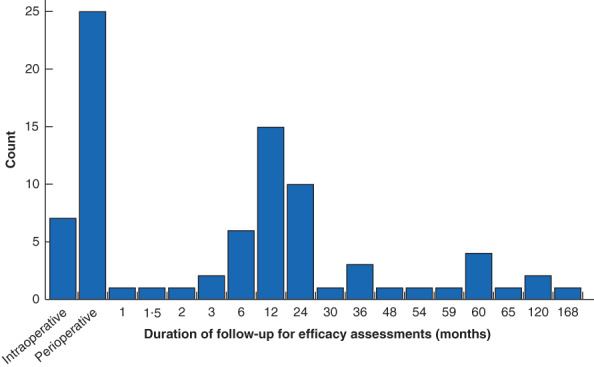
Frequency of follow‐up duration for the 83 studies that included efficacy assessment

**Fig. 6:**
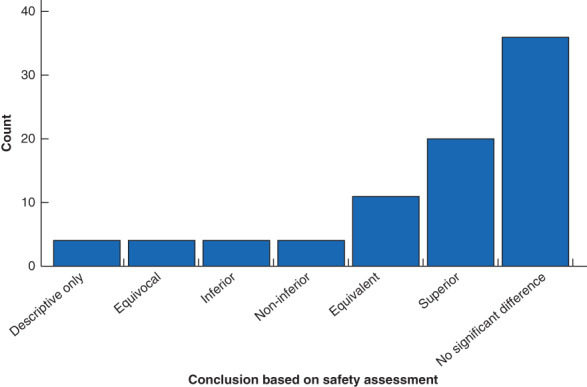
Conclusions based on efficacy parameters for the 83 studies that included efficacy assessment

### Efficacy or effectiveness assessments

Efficacy or effectiveness of robotic surgery was assessed in 80·6 per cent of the studies (83 of 103). Many studies reported multiple measures of effectiveness; these were often specialty‐ or procedure‐specific and not easily generalizable across procedure types. The commonest effectiveness assessments used a disease‐specific (38 of 83, 46 per cent) or general health (28 of 83, 35 per cent) PROM, lymph node retrieval (25 of 83, 30 per cent) and postoperative pain (24 of 83, 29 per cent). The follow‐up period tended to be longer for efficacy/effectiveness assessments than for safety assessments, with median of 12 (range 0–168) months where specified (*Fig*. *5*). Study conclusions based on efficacy assessments were most commonly found to show no significant difference (36 of 83, 43 per cent). Approximately one‐quarter of robotic procedures (20 of 83, 24 per cent) were reported to be more efficacious than their comparator group (*Fig*. *6*).

### Research governance

More than half of the studies (60 of 103, 58·3 per cent) reported a registration or record number with a recognized trial registry, but only around one‐quarter were registered prospectively (29 of 103, 28·2 per cent). In one publication the reported record number did not match the study performed. Funding declarations were present in 47·6 per cent (49 of 103) of published reports (or the online registration where available) and 54·4 per cent (56 of 103) included a conflict‐of‐interest statement.

## Discussion

This review identified a significant degree of duplicate publication from study populations and several publications from different stages of the same trial. Only 76 unique study populations were identified. Over one‐third of trials were not registered, and less than one‐third were registered prospectively. Many reported a different primary outcome to that in the trial protocol, had no declaration of funding, and no formal power calculation. This raises concerns about risk of bias, quality and transparency of research reporting across current randomized research in RAS. Urology and gynaecology were the commonest procedural types evaluated, and Intuitive Surgical's da Vinci® RAS platform was the commonest platform evaluated formally in included studies.

Outcomes evaluated in trials of RAS were numerous and varied. As with many clinical innovations, the best outcome measures for specific and extended applications are not always clear at the outset and are likely to evolve along with understanding of the platform. Often it appeared outcomes had been chosen for RAS studies because they were easy to measure rather than pointing to fundamental step‐changes in clinical care, or incremental benefit achieved in targeted and clinically meaningful variables^117^. Where a primary outcome measure was selected, it was infrequently registered prospectively, and studies were rarely powered to detect a difference in the measure.

Assessment of safety was commonly performed, highlighting that this was a key focus of RAS evaluation, with 68·9 per cent of RCTs addressing this area. The use of complications (99 per cent of those studies), conversion and secondary surgery may suggest a thinking that RAS can reduce any complication rate, or at least to a level equal to that of the current standard, if other benefits exist for RAS. Most safety outcomes were collected in the perioperative period, representing immediate or early sequelae of the surgical procedure. Blood loss requiring transfusion was measured in only 12 studies, and no studies assessed transfusion‐related complications. This is surprising as blood loss and transfusion is an immediate, easy to measure and consequential outcome. Major complications and mortality data were often proposed as markers of safety, but as these are rare and binary variables they are insensitive to detect differences between small groups of patients as seen in the included studies (median 78 patients).

The high rates of reporting effectiveness/efficacy outcomes (80·6 per cent of studies) highlighted the importance of evidence for comparative effectiveness studies in RAS for patients, surgeons, hospitals and industry. There was a far greater heterogeneity of outcomes reported for efficacy assessment than for safety, which made overall assessment of efficacy more complex. There may be a case for a core outcome set for robotic surgery in the future. Heterogeneity may also be a manifestation of the many specific settings and clinical needs of the patient groups. The use of pathology‐specific PROMs indicated that authors of the individual studies aimed to use validated measures that were important to patients, but no studies reported differences in terms of minimally clinically important differences or responsiveness^118^. Studies often analysed PROMs according to subscales of the score, driving up the number of outcomes reported and increasing the risk of a type I error. The lack of prospective registration and adequate powering of the studies added to this issue. No study reported on efforts to adjust for the impact of systems changes in their health network associated with developing a RAS service^21^, ^22^. Overall study conclusions based on efficacy measures were found most commonly to show no significant difference.

Other metrics seemed to be process or economically driven, such as duration of surgery and time to patient discharge. Duration of surgery offers little for safety or effectiveness/efficacy and can provide a perverse incentive. Half of the included studies looked for improvements in time to discharge, but this can be interpreted usefully only in the context of readmission rates, or whether the time saved improved theatre capacity to allow more procedures to be performed.

Given the limited intention to establish whether RAS is intrinsically safe or unsafe, effective or ineffective, it was still interesting to observe no overriding pattern around the safety and efficacy of RAS compared with other approaches. This review has highlighted that the evidence base remains small with poorly designed studies, including the striking overlap of sample populations used for different reported studies. Given this, any strong statements about safety and efficacy are largely unsupported. However, it should be noted, and is somewhat comforting, that the review did not uncover any clear evidence of surgical harms associated with RAS; this must be viewed in the context that the majority of these studies were not designed as non‐inferiority or equivalence trials, and heterogeneous outcomes prevent effective meta‐analysis.

Formal risk‐of‐bias assessment was not performed in this scoping review. No attempt was made to carry out meta‐analysis of safety or effectiveness measures. Instead, the review used surrogates for research quality and transparency, such as compliance with reporting guidelines^119^, conflicts of interest between researchers and manufacturers^120^, and statistical errors or misinterpretations^121^. These are facets of a pattern of poor research governance in RAS that have been studied specifically elsewhere^117^, ^119^, ^120^, ^121^, and the present findings concur with this pattern. The costs of RAS were not covered by this review, as these have been reported extensively^2^, ^21^, ^22^. Cost is an important metric for the evaluation of RAS because of high capital expenditure requirements; however, a more complete understanding of safety and effectiveness should precede a focus on cost. Cost‐effectiveness studies of RAS will be more meaningful after greater knowledge is gained of clinical outcomes, training and service delivery, and how these interact.

This review addressed only RCTs to ensure high‐quality evidence and generate an achievable number of studies for close assessment. It was also limited to the last decade in order to capture contemporary evidence (although the search was rerun before submission to capture additional studies published in the course of performing the review). Although RCTs have a high level of internal reliability and remain the standard for healthcare evaluation, they are difficult and costly to design, and cannot offer a sufficiently rapid evaluation, especially for a fast‐changing technology^6^. Caution should also be advised, as poorly designed RCTs can conflate the investment and infrastructure around developing a robotic surgical service without an independent element to the evaluation^60^, ^122^. Large database and more ‘real world’‐type studies with high engagement and fidelity may provide a more attractive option to detect and monitor longer‐term, rare outcomes or small effect sizes. However, such data can be prone to different systematic errors, such as selection bias^123^. Parallel initiatives involving both RCT and registry or hybrid designs (step wedge) should be considered.

The identification of risks and benefits associated with new surgical technologies from high‐quality evidence is critical. Research quality and the overall evidence base for RAS remains variable, with risk of bias. Outcome measures were many and varied, with scope for improved standardization. Reassuringly, no obvious patterns of threat or harm were identified; studies showed no difference between RAS and open or non‐robotic MIS in most cases, and significant benefit in some. The potential for RAS to be an integral component of the future of surgery remains high, but continued systematic and high‐quality evaluation is required.

## Supporting information


**Appendix** **S1** Search strategy
**Table S1** Principal studies used for analysis
