# Supplementary material for: Research quality and transparency, outcome measurement and evidence for safety and effectiveness in robot‐assisted surgery: systematic review
Source: BJS Open. 2020 Oct 14;4(6):1084–99. doi: 10.1002/bjs5.50352 (PMC7709372; doi:10.1002/bjs5.50352)
Supplement: Supplementary file 1 — Appendix S1 Search strategy Table S1 Principal studies used for analysis [file BJS5-4-1084-s001.docx]

**BJS5_50352**

**Research quality and transparency, outcome measurement and evidence for safety and effectiveness in robot-assisted surgery: systematic review**

**P. Garfjeld Roberts, J. C. Glasbey, S. Abram, D. Osei-Bordom, S. P. Bach and D. J. Beard**

## **Appendix S1** Search strategy

With thanks to Eli Harriss, Librarian at the University of Oxford

Database: Medline (Ovid MEDLINE® Epub Ahead of Print, In-Process & Other Non-Indexed Citations, Ovid MEDLINE® Daily

and Ovid MEDLINE®) 1946 to present

Search Strategy:

--------------------------------------------------------------------------------

1 Robotics/ or Robotic Surgical Procedures/

2 exp Surgical Procedures, Operative/

3 exp Specialties, Surgical/

4 2 or 3

5 1 and 4

6 Robotic Surgical Procedures/

7 (robot* and (gastrotomy or arthroplasty or prostatectomy or nephrectomy or hysterectomy or operati* or procedure* or resection* or surg*)).ti,ab.

8 5 or 6 or 7

9 randomised.ti,ab.

10 randomized.ti,ab.

11 randomly.ti,ab.

12 placebo.ti,ab.

13 trial.ti.

14 exp Clinical Trials as Topic/

15 randomized controlled trial.pt.

16 controlled clinical trial.pt.

17 9 or 10 or 11 or 12 or 13 or 14 or 15 or 16

18 8 and 17

19 18

20 limit 19 to (english language and yr="2008 - 2019")

**Database: Embase 1974 to present**

Search Strategy:

--------------------------------------------------------------------------------

1 robotics/

2 robotic surgical procedure/

3 1 or 2

4 exp surgery/

5 3 and 4

6 (robot* and (gastrotomy or arthroplasty or prostatectomy or nephrectomy or hysterectomy or operati* or procedure* or resection* or surg*)).ti,ab.

7 2 or 5 or 6

8 crossover procedure/

9 double blind procedure/

10 randomized controlled trial/

11 single blind procedure/

12 random*.mp.

13 factorial*.mp.

14 (crossover* or cross over* or "cross‐over*").mp.

15 placebo*.mp.

16 (double* adj blind*).mp.

17 (singl* adj blind*).mp.

18 assign*.mp.

19 allocat*.mp.

20 volunteer*.mp.

21 8 or 9 or 10 or 11 or 12 or 13 or 14 or 15 or 16 or 17 or 18 or 19 or 20

22 7 and 21

23 22

24 limit 23 to (english language and yr="2008 - 2019")

**Cochrane Central Register of Controlled Trials**

#1 MeSH descriptor: [Robotics] explode all trees

#2 MeSH descriptor: [Robotic Surgical Procedures] explode all trees

#3 #1 or #2

#4 MeSH descriptor: [Surgical Procedures, Operative] explode all trees

#5 MeSH descriptor: [Specialties, Surgical] explode all trees

#6 #4 or #5

#7 #3 and #6

#8 (robot* and (gastrotomy or arthroplasty or prostatectomy or nephrectomy or hysterectomy or operati* or procedure* or resection* or surg*))

#9 #2 or #7 or #8

Filter your results: 2008 to 2019

**Scopus**

( TITLE-ABS-KEY ( robot* AND ( gastrotomy OR arthroplasty OR prostatectomy OR nephrectomy OR hysterectomy OR operati* OR procedure* OR resection* OR surg* ) ) ) AND ( ( TITLE-ABS-KEY ( randomised OR randomized OR randomly ) ) OR ( TITLE-ABS-KEY ( placebo ) ) OR ( TITLE ( trial ) ) OR ( TITLE-ABS-KEY ( rct* ) ) OR ( TITLE-ABS-KEY ( crossover* OR cross AND over* OR "cross‐over*" OR assign* OR allocat* OR volunteer* ) ) ) AND ( LIMIT-TO ( PUBYEAR , 2019 ) OR LIMIT-TO ( PUBYEAR , 2018 ) OR LIMIT-TO ( PUBYEAR , 2017 ) OR LIMIT-TO ( PUBYEAR , 2016 ) OR LIMIT-TO ( PUBYEAR , 2015 ) OR LIMIT-TO ( PUBYEAR , 2014 ) OR LIMIT-TO ( PUBYEAR , 2013 ) OR LIMIT-TO ( PUBYEAR , 2012 ) OR LIMIT-TO ( PUBYEAR , 2011 ) OR LIMIT-TO ( PUBYEAR , 2010 ) OR LIMIT-TO ( PUBYEAR , 2009 ) OR LIMIT-TO ( PUBYEAR , 2008 ) ) AND ( LIMIT-TO ( LANGUAGE , "English" ) )

**Web of Science Core Collection**

1. TITLE: (robot* near/8 (gastrotomy or arthroplasty or prostatectomy or nephrectomy or hysterectomy or operati* or procedure* or resection* or surg*))
2. TOPIC: (randomised OR randomized OR randomly OR placebo)
3. TOPIC: (trial OR RCT*)
4. #2 or #3
5. #1 AND #4
6. #5 Refined by: PUBLICATION YEARS: ( 2019 OR 2018 OR 2011 OR 2017 OR 2010 OR 2016 OR 2009 OR 2015 OR 2008 OR 2014 OR 2013 OR 2012 ) AND LANGUAGES: ( ENGLISH )

## **Table S1** Principal studies used for analysis

| Study | Specialty | Pathology addressed | Robotic device | Comparison groups | Total participants | Safety assessment | Efficacy assessment | Number of outcomes in study |
| --- | --- | --- | --- | --- | --- | --- | --- | --- |
| Abdalla 2017^14^  Hernia | Colorectal | Incisional hernia | Not known | 2 | 38 | + | + | 5 |
| Anger 2014^15^  Obstetrics & Gynecology | ObsGynae | Pelvic organ prolapse | Not known | 2 | 78 | + | + | 19 |
| Ashwin 2014^16^  European Journal of Surgical Oncology | ObsGynae | Endometrial Ca | Da Vinci (Intuitive) | 2 | 118 | + | + | 5 |
| Asimakopoulos 2011^17^  Journal of Sexual Medicine | Urology | Ca Prostate | Da Vinci (Intuitive) | 2 | 128 | + | + | 27 |
| Baik 2008^18^  Surgical Endoscopy and Other Interventional Techniques | Colorectal | Ca Rectum | Da Vinci (Intuitive) | 2 | 36 | + | + | 11 |
| Bargar 2018^19^  Journal of Arthroplasty | T&O | Osteoarthritis - hip | Robodoc (Think Surgical) | 2 | 125 | - | + | 11 |
| Bell 2016^20^  Journal of Bone and Joint Surgery - American Volume | T&O | Osteoarthritis - medial compartment of knee | Robotic Arm Interactive Orthopaedic System (MAKO) | 2 | 139 | - | + | 12 |
| Bhattu 2015^21^  Journal of Endourology | Urology | Kidney donation | Da Vinci (Intuitive) | 2 | 45 | + | + | 45 |
| Blyth 2017^22^  Bone & Joint Research | T&O | Osteoarthritis - medial compartment of knee | Robotic Arm Interactive Orthopaedic System (MAKO) | 2 | 139 | - | + | 15 |
| Bochner 2015^23^  European Urology | Urology | Ca Bladder | Not known | 2 | 118 | + | + | 40 |
| Bochner 2018^24^  European Urology | Urology | Ca Bladder | Not known | 2 | 118 | - | + | 8 |
| Calza 2011^25^  Journal of Urology | Urology | Ca Prostate | Not known | 2 | 120 | - | - |  |
| Chen 2017^26^  Surgical Endoscopy | Upper GI | Pancreatic pathology requiring pancreatectomy (benign/low-grade malignant) | Da Vinci (Intuitive) | 2 | 107 | + | - | 8 |
| Coughlin 2018^27^  Lancet Oncology | Urology | Ca Prostate | Not known | 2 | 326 | - | + | 13 |
| Debakey 2018^28^  Minimally Invasive Surgery | Colorectal | Ca Rectum | Da Vinci (Intuitive) | 2 | 57 | + | + | 12 |
| Deimling 2016^29^  International Journal of Gynecology and Obstetrics | ObsGynae | Hysterectomy | Da Vinci (Intuitive) | 2 | 144 | + | - | 18 |
| DeRooij 2017^30^  United European Gastroenterology Journal (Conference) | Upper GI | Pancreatic pathology requiring pancreatectomy (benign/low-grade malignant) | Not known | 2 | 108 | + | + | 11 |
| Feng 2019^31^  World Neurosurgery | T&O | Lumbosacral spinal disorders | TiRobot (TiNavi Technologies) | 2 | 80 | - | + | 10 |
| Gargini 2009^32^  Gynecological Surgery | ObsGynae | Ca Cervix | Not known | 2 | 16 | + | + | 8 |
| Geavlete 2016^33^  Journal of Endourology | Urology | Urinary tract stones | Avicenna Roboflex (Elmed-Turkey) | 2 | 132 | + | + | 6 |
| Giberti 2017^34^  Canadian Journal of Urology | Urology | Ca Prostate | Not known | 2 | 83 | - | + | 3 |
| Gilling 2018^35^  Journal of Urology | Urology | BPH | AquaBeam (Procept Biorobotics) | 2 | 184 | + | + | 25 |
| Gilling 2019^36^  Advances in therapy | Urology | BPH | Not known | 2 | 184 | - | + | 22 |
| Gilmour 2018^37^  Journal of Arthroplasty | T&O | Osteoarthritis - medial compartment of knee | Robotic Arm Interactive Orthopaedic System (MAKO) | 2 | 139 | + | + | 14 |
| Grochola 2018^38^  Surgical Endoscopy | Upper GI | Benign gallbladder disease | Da Vinci (Intuitive) | 2 | 60 | + | + | 11 |
| Han 2019^39^  Journal of Neurosurgery: Spine | T&O | Thoracolumbar spinal disorders | TiRobot (TiNavi Technologies) | 2 | 234 | - | + | 12 |
| Hyun 2017^40^  Spine | T&O | Degenerative spinal disorders | Renaissance (Mazor) | 2 | 60 | + | + | 13 |
| Illiano 2018^41^  Neurourology and Urodynamics | ObsGynae | Pelvic organ prolapse | Not known | 2 | 71 | + | + | 6 |
| Iyigun 2017^42^  International Journal of Surgery | Cardiac | Cardiac pathology requiring median sternotomy | Da Vinci (Intuitive) | 2 | 62 | - | - | 7 |
| Jayne 2017^43^  JAMA | Colorectal | Ca Rectum | Not known | 2 | 471 | + | + | 8 |
| Jimenez Rodriguez 2011^44^  Cirugia espanola | Colorectal | Right-sided colorectal Ca | Not known | 2 | 56 | + | + | 14 |
| Jin 2019^45^  Chest | Thoracic | Lung Ca | Not known | 2 | 139 | - | + | 4 |
| Kenton 2013^46^  Journal of Urology | ObsGynae | Pelvic organ prolapse | Not known | 2 | 78 | - | - | 9 |
| Kenton 2016^47^  Female Pelvic Medicine & Reconstructive Surgery | ObsGynae | Pelvic organ prolapse | Not known | 2 | 78 | - | + | 11 |
| Khan 2016^48^  European Urology | Urology | Ca Bladder | Da Vinci (Intuitive) | 3 | 60 | + | + | 12 |
| Kim 2017^49^  Spine Journal | T&O | Lumbar spinal stenosis | Renaissance (Mazor) | 2 | 78 | - | - | 6 |
| Kim 2017^50^  The International Journal Of Medical Robotics + Computer Assisted Surgery: MRCAS | T&O | Lumbar spinal stenosis | Renaissance (Mazor) | 2 | 78 | + | + | 24 |
| Kim 2018^51^  Annals of Surgery | Colorectal | Ca Rectum | Da Vinci (Intuitive) | 2 | 163 | + | + | 43 |
| Kim 2018^52^  The International Journal Of Medical Robotics + Computer Assisted Surgery: MRCAS | T&O | Lumbar spinal stenosis | Renaissance (Mazor) | 2 | 78 | - | + | 6 |
| Kim 2019^53^  Clinical Orthopaedics & Related Research | T&O | Osteoarthritis of knee | Orthodoc (Integrated Surgical Technology) | 2 | 1516 | + | + | 31 |
| Kudsi 2017^54^  Surgical Endoscopy | Upper GI | Benign gallbladder disease | Da Vinci (Intuitive) | 2 | 136 | + | + | 10 |
| Lim 2015^55^  Computer Aided Surgery | T&O | Osteoarthritis - hip | Robodoc (Think Surgical) | 2 | 54 | + | + | 15 |
| Lim 2017^56^  International Journal of Computer Assisted Radiology and Surgery | T&O | Osteoarthritis - hip | Robodoc (Think Surgical) | 2 | 54 | + | + | 3 |
| Liow 2014^57^  Journal of Arthroplasty | T&O | Osteoarthritis of knee | Robodoc (Think Surgical) | 2 | 60 | + | + | 40 |
| Liow 2017^58^  Knee Surgery, Sports Traumatology, Arthroscopy | T&O | Osteoarthritis of knee | Robodoc (Think Surgical) | 2 | 60 | - | + | 29 |
| Lonnerfors 2015^59^  Journal of Minimally Invasive Gynecology | ObsGynae | Hysterectomy | Da Vinci (Intuitive) | 2 | 122 | + | - | 19 |
| Lundin 2019^60^  International Journal of Gynecological Cancer | ObsGynae | Endometrial Ca | Da Vinci (Intuitive) | 2 | 50 | + | + | 19 |
| Luo 2018^61^  BMC Women's Health | ObsGynae | Ca Cervix | Da Vinci (Intuitive) | 2 | 60 | + | + | 4 |
| Luu 2016^62^  Fertility and Sterility | ObsGynae | Endometriosis | Da Vinci (Intuitive) | 2 | 73 | - | - | 1 |
| Maenpaa 2016^63^  American Journal of Obstetrics & Gynecology | ObsGynae | Endometrial Ca | Da Vinci (Intuitive) | 2 | 101 | + | + | 18 |
| Makela-Kaikkonen 2016^64^  Colorectal Disease | ObsGynae | Pelvic organ prolapse | Da Vinci (Intuitive) | 2 | 30 | + | + | 15 |
| Makela-Kaikkonen 2016^65^  International Urogynecology Journal | ObsGynae | Pelvic organ prolapse | Da Vinci (Intuitive) | 2 | 30 | - | + | 33 |
| Makela-Kaikkonen 2019^66^  Techniques in Coloproctology | ObsGynae | Pelvic organ prolapse | Da Vinci (Intuitive) | 2 | 30 | - | + | 48 |
| Materazzi 2014^67^  World Journal of Surgery | Endocrine | Benign thyroid nodule | Da Vinci (Intuitive) | 2 | 62 | + | + | 15 |
| Milanez 2012^68^  Innovations: Technology and Techniques in Cardiothoracic and Vascular Surgery | Cardiac | Cardiac bypass | Aesop (Computer Motion) | 2 | 36 | + | + | 5 |
| Motesharei 2018^69^  Gait & Posture | T&O | Osteoarthritis - medial compartment of knee | Robotic Arm Interactive Orthopaedic System (MAKO) | 2 | 70 | - | + | 4 |
| Mueller 2016^70^  Journal of Minimally Invasive Gynecology | ObsGynae | Pelvic organ prolapse | Not known | 2 | 78 | - | - | 3 |
| Muller-Stich 2009^71^  Langenbeck's archives of surgery | Upper GI | Gastro-oesophageal reflux disease | Da Vinci (Intuitive) | 2 | 40 | - | + | 18 |
| Nakamura 2010^72^  Clinical Orthopaedics & Related Research | T&O | Osteoarthritis - hip | Robodoc (Think Surgical) | 2 | 146 | + | + | 13 |
| Nakamura 2018^73^  Clinical Orthopaedics & Related Research | T&O | Osteoarthritis - hip | Robodoc (Think Surgical) | 2 | 146 | - | + | 4 |
| Narducci 2017^74^  International Journal of Gynecological Cancer (Conference) | ObsGynae | Ca uterine/cervical/ovarian | Not known | 2 | 369 | + | - | 1 |
| Nichols 2019^75^  Lancet Oncology | ENT | Oropharyngeal squamous cell carcinoma | Not known | 2 | 34 | + | + | 11 |
| Nix 2010^76^  European Urology | Urology | Ca Bladder | Da Vinci (Intuitive) | 2 | 41 | + | + | 9 |
| Omar 2018^77^  Journal of Clinical Urology | Urology | Ca Bladder | Da Vinci (Intuitive) | 3 | 60 | - | + | 6 |
| Pan 2017^78^  Surgical Laparoscopy, Endoscopy & Percutaneous Techniques | Upper GI | Ca Gastric | Not known | 2 | 163 | + | - | 10 |
| Paraiso 2011^79^  Obstetrics & Gynecology | ObsGynae | Pelvic organ prolapse | Da Vinci (Intuitive) | 2 | 78 | + | + | 26 |
| Paraiso 2013^80^  American Journal of Obstetrics and Gynecology | ObsGynae | Hysterectomy | Da Vinci (Intuitive) | 2 | 62 | + | + | 19 |
| Parekh 2018^81^  Lancet | Urology | Ca Bladder | Not known | 2 | 350 | + | + | 28 |
| Park 2012^82^  British Journal of Surgery | Colorectal | Right-sided colorectal Ca | Da Vinci (Intuitive) | 2 | 71 | + | + | 17 |
| Park 2018^83^  Surgical Endoscopy | Colorectal | Right-sided colorectal Ca | Da Vinci (Intuitive) | 2 | 71 | + | + | 13 |
| Park 2018^84^  Yonsei Medical Journal | T&O | Lumbar spinal stenosis | Renaissance (Mazor) | 2 | 78 | - | + | 8 |
| Passerotti 2011^85^  European Urology, Supplements | Urology | Ca Prostate | Not known | 2 | 200 | - | + | 5 |
| Pietrabissa 2016^86^  Surgical Endoscopy | Upper GI | Benign gallbladder disease | Da Vinci (Intuitive) | 2 | 81 | + | - | 6 |
| Plante 2018^87^  BJU International | Urology | BPH | AquaBeam (Procept Biorobotics) | 2 | 181 | + | + | 46 |
| Porpiglia 2013^88^  European Urology | Urology | Ca Prostate | Not known | 2 | 120 | + | + | 10 |
| Porpiglia 2018^89^  European Urology Focus | Urology | Ca Prostate | Not known | 2 | 120 | + | + | 10 |
| Ramirez 2018^90^  New England Journal of Medicine | ObsGynae | Ca Cervix | Not known | 2 | 631 | - | + | 3 |
| Ringel 2012^91^  Spine | T&O | Benign surgical spinal pathology | Spine Assist (Mazor) | 2 | 60 | - | + | 8 |
| Roser 2013^92^  Neurosurgery | T&O | Degenerative spinal disorders | Spine Assist (Mazor) | 3 | 148 | + | + | 7 |
| Salehi 2017^93^  European Journal of Cancer | ObsGynae | Endometrial Ca | Da Vinci (Intuitive) | 2 | 120 | + | + | 10 |
| Sarlos 2012^94^  Obstetrics & Gynecology | ObsGynae | Hysterectomy | Da Vinci (Intuitive) | 2 | 100 | + | + | 19 |
| Silay 2019^95^  World Journal of Urology | Paediatric | Ureteropelvic junction obstruction | Da Vinci (Intuitive) | 2 | 53 | + | + | 21 |
| Silva 2018^96^  Clinics (Sao Paulo, Brazil) | ObsGynae | Endometrial Ca | Da Vinci (Intuitive) | 2 | 85 | + | + | 7 |
| Somashekhar 2014^97^  Indian Journal of Surgical Oncology | ObsGynae | Endometrial Ca | Da Vinci (Intuitive) | 2 | 50 | + | + | 11 |
| Somashekhar 2015 ^98^  Indian Journal of Surgery | Colorectal | Ca Rectum | Da Vinci (Intuitive) | 2 | 50 | + | + | 8 |
| Song 2011^99^  Knee Surgery Sports Traumatology Arthroscopy | T&O | Osteoarthritis of knee | Robodoc (Think Surgical) | 2 | 60 | - | + | 17 |
| Song 2013^100^  Clinical Orthopaedics & Related Research | T&O | Osteoarthritis of knee | Robodoc (Think Surgical) | 2 | 100 | + | + | 19 |
| Soto 2017^101^  Fertility and Sterility | ObsGynae | Endometriosis | Da Vinci (Intuitive) | 2 | 73 | + | + | 4 |
| Tarnay 2013^102^  Neurourology and Urodynamics | ObsGynae | Pelvic organ prolapse | Not known | 2 | 78 | - | - | 3 |
| Tolstrup 2018^103^  International Journal of Colorectal Disease | Colorectal | Ca Rectum | Not known | 2 | 51 | + | + | 15 |
| Tzvetanov 2015^104^  American Journal of Transplantation (Conference) | Transplant | Obesity with renal failure | Not known | 2 | 4 | + | + | 3 |
| Udell 2012^105^  Journal of Urology | Urology | Ca Bladder | Da Vinci (Intuitive) | 2 | 41 | + | + | 4 |
| Udell 2012^106^  Journal of Urology | Urology | Ca Bladder | Da Vinci (Intuitive) | 2 | 41 | - | - | 3 |
| Van der Sluis 2018^107^  Annals of Surgery | Upper GI | Ca Oesophagus | Da Vinci (Intuitive) | 2 | 112 | + | + | 69 |
| Wagner 2019^108^  European Urology, Supplements | ObsGynae | Pelvic organ prolapse | Da Vinci (Intuitive) | 2 | 345 | + | - | 86 |
| Wang 2016^109^  Journal of Surgical Oncology | Upper GI | Ca Gastric | Not known | 2 | 311 | + | + | 7 |
| Wang 2017^110^  Chinese Medical Journal | T&O | Pelvic ring posterior instability | TiRobot (TiNavi Technologies) | 2 | 30 | + | + | 4 |
| Wijk 2018^111^  Clinical Nutrition | ObsGynae | Total hysterectomy and/or bilateral salpingo-oophorectomy for benign or malignant indications | Da Vinci (Intuitive) | 2 | 20 | + | - | 19 |
| Wurnschimmel 2019^112^  European Urology, Supplements | Urology | Renal Ca | Not known | 2 | 74 | + | + | 8 |
| Xu 2017^113^  Annals of Oncology | Colorectal | Ca Rectum | Da Vinci (Intuitive) | 3 | 506 | + | + | 11 |
| Xu 2017^114^  Annals of Oncology | Colorectal | CRCLM (Colorectal cancer with liver mets) | Not known | 2 | 120 | + | - | 4 |
| Yaxley 2016^115^  The Lancet | Urology | Ca Prostate | Not known | 2 | 326 | + | + | 34 |
| Zhou 2019^116^  International Journal of Clinical and Experimental Medicine | Urology | Renal Ca | Not known | 2 | 75 | + | - | 26 |
